# Low frequency of male circumcision and unwillingness to be circumcised among MSM in Buenos Aires, Argentina: association with sexually transmitted infections

**DOI:** 10.7448/IAS.16.1.18500

**Published:** 2013-06-06

**Authors:** María A Pando, Ivan C Balan, Curtis Dolezal, Ruben Marone, Victoria Barreda, Alex Carballo-Dieguez, María M Avila

**Affiliations:** 1 Instituto de Investigaciones Biomédicas en Retrovirus y SIDA (INBIRS), Facultad de Medicina, Universidad de Buenos Aires, Buenos Aires, Argentina; 2 Consejo Nacional de Investigaciones Científicas y Técnicas (CONICET), Buenos Aires, Argentina; 3 HIV Center for Clinical and Behavioral Studies, New York State Psychiatric Institute and Columbia University, New York, NY, USA; 4 Nexo Asociación Civil, Buenos Aires, Argentina

**Keywords:** men who have sex with men, circumcision, respondent-driven sampling, HPV, HIV, Buenos Aires

## Abstract

**Objective:**

The aims of this study were to investigate the frequency of male circumcision among men who have sex with men (MSM) in Buenos Aires, Argentina; the association between circumcision and sexually transmitted infections (STIs); and, among those uncircumcised, the willingness to be circumcised.

**Methods:**

A cross-sectional study was conducted among 500 MSM recruited through the respondent-driven sampling (RDS) technique. Participants underwent a consent process, responded to a Web-based survey that included questions on demographic information, sexual behaviour, and circumcision and provided biological samples. HIV, hepatitis B virus (HBV), hepatitis C virus (HCV), *Treponema pallidum*, and human papiloma virus (HPV) diagnoses were performed using standard methodologies. For all analyses, data were weighted based on participants’ network size.

**Results:**

Only 64 (13%) of the 500 MSM in our study reported being circumcised. Among uncircumcised men (*n*=418), 302 (70.4%) said that they would not be willing to get circumcised even if the procedure could reduce the risk of HIV infection. When considering all participants, circumcision status was not significantly associated with HIV, HBV, HCV, *T. pallidum* or HPV infections. However, when we restricted the sample to men who do not practice receptive anal intercourse (RAI) and compared circumcised to uncircumcised men, the former (*N*=33) had no cases of HIV infection, while 34 of 231 (14.8%) uncircumcised men were HIV positive (*p*=0.020). Regarding HPV, uncircumcised men had a significantly larger number of different HPV types compared with circumcised men (mean 1.83 vs. 1.09, *p<*0.001) and a higher frequency of high-risk-HPV genotypes (47.6% vs. 12.5%, *p*=0.012).

**Conclusions:**

Consistent with international evidence, male circumcision appears to have a partial protective effect among MSM. The efficacy of circumcision in reducing risk of HIV infection among MSM appears to be correlated with sexual practices. Given the lack of motivation among MSM with regard to circumcision, proper awareness on the risks and benefits of circumcision needs to be created, if circumcision has to be introduced as a prevention strategy.

## Introduction

Although male circumcision has been associated with religious practices and ethnicity, in the 20th century the procedure was introduced for health reasons. An estimated 30% of males are circumcised globally, the practice being more common in the Middle East, Central Asia and West Africa. In the United States, Canada, Australia and New Zealand, circumcision is common but not related to religious practices [1]. A survey among 18 countries from Central and South America (not including Argentina) revealed that fewer than 20% of the males are circumcised [2].

Three trials among heterosexual men demonstrated that circumcision reduces HIV acquisition by 60% [3–5]. Ecological studies also showed that HIV prevalence among both men and women is lower in countries with high levels of male circumcision [6]. There is also evidence that circumcised men are at lower risk of human papilloma virus (HPV) [7] and herpes simplex [8]. In this context, the Joint United Nations Programme on HIV and AIDS (UNAIDS) recommended that circumcision be added to the list of HIV prevention strategies for heterosexual men [9, 10].

The potential efficacy of circumcision in reducing HIV infection among men who have sex with men (MSM) is unclear. Most studies have not found an association between HIV prevalence and incidence, on one hand, and circumcision, on the other, among MSM [11] because of multiple factors, including evidence that circumcision will not reduce HIV acquisition through unprotected receptive anal intercourse (RAI). However, studies of MSM who predominantly practice insertive anal intercourse (IAI) suggest some protective effect as reported in Peru [12], South Africa [13] and Australia [14].

Studies performed in Argentina revealed that MSM are at high risk of HIV infection [15–17]. Prevalence of HIV has been estimated most recently at 17.3% and incidence at 4.53 per 100 person-years. Other sexually transmitted infections (STIs) also appear to be highly prevalent among MSM: hepatitis B (HBV; 22.9%), hepatitis C (HCV; 7.5%), *Treponema pallidum* (20.5%) and HPV (83.5%) [18].

No studies have explored the frequency of male circumcision in Argentina or the influence of circumcision on HIV acquisition. Given that this information is essential to planning prevention programmes, the aims of this study were to investigate the frequency of male circumcision among MSM in Buenos Aires, Argentina; the association between circumcision and STIs; and, among those uncircumcised, the willingness to be circumcised.

## Methods

### Study population and procedures

ProyectoLINKS was a cross-sectional study with the objective of assessing HIV prevalence and incidence among MSM in Buenos Aires, Argentina. Participants were recruited through respondent-driven sampling (RDS) between November 2007 and July 2009. Recruitment methodology and data collection have been previously described [19]. Briefly, 16 MSM who were selected as seeds for the RDS completed all study procedures and received three coupons each to be given to members of their networks. All participants were offered a dual incentive (for participation and for each eligible person recruited). At the end of the interview, each participant received the equivalent of 20 USD (equivalent to the cost of five movie tickets) as compensation for his time. For each referred acquaintance who qualified for the study, regardless of whether he enrolled or not, the participant received an additional USD 5.

To be eligible, a study candidate had to identify as a man, be 18 years or older, have had sex with another man or a trans person (transvestite, transgender or transsexual) at least once in the past six months and at least ten times in his lifetime, reside in the Buenos Aires metropolitan area, agree to provide a blood sample for STI testing, and come to the interview with a coupon given by a prior participant. Participants underwent a consent process and responded to a Web-based survey that included questions on demographic information, sexual behaviour and circumcision, among others. Subsequently, participants received HIV pre-test counselling and biological samples were collected for STI testing (HIV, HBV, HCV, HPV, *T. pallidum* and *C. trachomatics*) [18]. Two weeks later, participants returned to obtain results and receive post-test counselling.

International and national ethical guidelines for biomedical research involving human subjects were followed. This study received approval from a local Institutional Review Board (IRB) (Facultad de Medicina, Universidad de Buenos Aires) and the IRB of the New York State Psychiatric Institute.

### Sample collection and STI diagnoses

A sample of anticoagulated blood was collected for determination of HIV, HBV, HCV and *T. pallidum* infection as previously described [18]. Tests for HPV and *C. trachomatis* infection were offered to all participants. Those who accepted were instructed on how to use an anal brush to obtain the sample. Samples were then processed as previously described [18].

### Sample size calculation

HIV prevalence data from prior studies [15, 20] were used to determine the sample size necessary to provide a precise estimate of HIV. It was determined that a sample size of 250 would be sufficient for most associations, and, using a design effect of 2.0 (oversampling suggested when using RDS by Heckathorn, 1997) [21], an adjusted sample size of 500 was determined for this study.

### Measures

As part of the Web-based survey, participants were asked: 1) if they were circumcised; and, for those who said no, 2) their willingness to be circumcised if that could protect them against HIV and their reasons to do or not to do it (open answers). As part of the sexual behaviour assessment, participants were asked about the gender of their sex partners (man, woman or trans person) and frequency of RAI and/or IAI. Gender of sexual partners was asked, referring to the prior year, and sexual behaviours were restricted to the prior two months to increase accuracy of recall.

### Statistical analysis

Data were weighted prior to analyses using SPSS (version 20; SPSS Inc., Chicago, IL, USA). Weights were calculated as the inverse of the participant's personal network size (PNS). This value was then multiplied by the sample size (*N*) divided by the sum of weights (Σw). The weighting formula is then:

(1/PNS)*(N/Σw).

This produces results that reflect the original sample size of 500. All results presented in this manuscript are based on weighted data. Circumcised and uncircumcised men were compared using a *t*-test for one normally distributed continuous variable (age), Mann-Whitney tests for continuous variables with skewed distributions (partner number variables), and Fisher's exact tests for dichotomous variables (sexual practices and STIs).

## Results

### Study population characteristics

A total of 500 MSM were recruited. Participants were young (mean age=30.5 years, standard deviation=11.5), they were mostly unemployed or only temporarily employed (30% and 32%, respectively), and many (66%) did not have a high school degree. Most participants were single (78%) and lacked health insurance (79%).

### Frequency of circumcision

Among the 500 MSM included in the study, 482 reported their circumcision status. Only 64 (13%) stated that they were circumcised. As shown in Table 1, there were no significant differences between circumcised and uncircumcised men in terms of demographic (age, formal education level, employment, and health insurance) or sexual behaviour characteristics (type and number of partners, receptive and insertive practices, and unprotected intercourse).

**Table 1:** Demographic and behavioural characteristics among MSM (*n*=482) recruited through response-driven sampling, Buenos Aires, Argentina, 2007–2009

	Circumcised *N* (%)	Uncircumcised *N* (%)	*p*
*N* (%)	64 (13.4)	418 (86.6)	
Age (years)^a^	25 [20–38]	28 [21–39]	0.159
Completed high school	28 (43.8)	136 (32.9)	0.091
Unemployed	20 (30.8)	129 (30.9)	1.000
Without health insurance	46 (70.8)	330 (79.7)	0.107
Sexual partners^b^			
Only men	19 (31.7)	102 (26.1)	0.353
Men and women and/or trans	41 (68.3)	289 (73.9)	
Male partners (*n*=438)^b^			
Number of partners^a^	2 [1–8]	2 [1–6]	0.789
Any RAI	26 (43.3)	144 (39.8)	0.670
Any URAI	14 (23.3)	73 (20.2)	0.606
Any IAI	52 (86.7)	320 (88.4)	0.669
Any UIAI	27 (45.0)	153 (42.3)	0.778
Female partners (*n*=313)^b^			
Number of partners^a^	4 [2–11]	3 [1–5]	0.254
Any IVI	34 (100.0)	266 (99.6)	1.000
Any UIVI	26 (76.5)	158 (59.2)	0.062
Any IAI	28 (80.0)	206 (77.2)	0.831
Any UIAI	20 (57.1)	121 (45.3)	0.210
Trans partners (*n*=226)^b^			
Number of partners^a^	2 [1–4]	2 [1–3]	0.810
Any RAI	6 (18.2)	27 (14.4)	0.597
Any URAI	3 (9.1)	14 (7.5)	0.725
Any IAI	31 (93.9)	168 (89.8)	0.748
Any UIAI	12 (37.5)	62 (33.0)	0.687

a Median [interquartile range].

b Data from the past two months; number of partners and sexual practices are limited to men with the concerned partner type.

MSM, men who have sex with men; RAI, receptive anal intercourse; URAI, unprotected anal intercourse; IAI, insertive anal intercourse; UIAI, unprotected insertive anal intercourse; IVI, insertive vaginal intercourse; UIVI, unprotected insertive vaginal intercourse.Due to missing data, sample size may differ.

### Association between male circumcision and STIs

When considering the entire group of MSM, circumcision was not associated with HIV, HBV, HCV, *T. pallidum* or HPV infections (Table 2). However, stratifying the group according to their sexual role in the past two months and restricting the analysis to those who do not practice RAI, no cases of HIV infection were detected among circumcised men (*N*=33), while 14.8% (34 of 231) of uncircumcised men were HIV positive (*p*=0.020).

**Table 2:** Association between STIs and circumcision among MSM (*n*=482) recruited through response-driven sampling, Buenos Aires, Argentina, 2007–2009, with stratification according to receptive anal intercourse practices

	Total MSM (*n*=482)			MSM not engaged in RAI^a^ (*N*=264)			MSM engaged in RAI^a^ (*N*=185)		
			
Prevalence	Circumcised (*n*=64)	Uncircumcised (*n*=418)	*p*	Circumcised (*n*=33)	Uncircumcised (*n*=231)	*p*	Circumcised (*n*=31)	Uncircumcised (*n*=154)	*p*
HIV	10.9	17.8	0.211	0.0	14.8	**0.020**	22.6	24.0	1.000
HBV	24.6	22.0	0.633	15.6	18.3	1.000	32.3	28.6	0.670
HCV	1.6	8.4	0.069	3.0	11.3	0.219	0.0	3.9	0.592
*T. pallidum*	25.0	19.9	0.325	25.0	13.4	0.108	22.6	30.3	0.516
HPV	88.9	81.2	0.733	75.0	85.7	0.527	92.3	79.7	0.442

a RAI with men and/or trans.

MSM, men who have sex with men; RAI, receptive anal intercourse; HIV, human immunodeficiency virus; HBV, hepatitis B; HCV, hepatitis C; HPV, human papillomavirus. Statistically significant associations are illustrated in boldface.

No significant association was observed between circumcision and infection for HBV, HCV, *T. pallidum* and HPV, even after stratifying the group according to the practice of RAI. Regarding HPV infection, uncircumcised men had a significantly larger number of different HPV types compared with circumcised men (mean 1.83 vs. 1.09, *p<*0.001) and a higher frequency of high-risk-HPV genotypes (47.6% vs. 12.5%, *p*=0.012).

### Willingness to be circumcised

Among uncircumcised men (*n*=418), 75% said that they would not be willing to be circumcised even if it could be beneficial in reducing risk of HIV infection. Among the remaining men, 13% expressed being “somewhat willing,” 5% were “very willing” and 8% were “completely willing” to be circumcised. The reasons for unwillingness to be circumcised included aesthetic reasons and the feeling that it is a mutilation of the body (30%), doubts about its effectiveness (25%), fear of surgery (21%), lack of interest (13%) and possibility of diminished sexual sensation (3%).

## Discussion

This study explored, for the first time in Argentina, the frequency of male circumcision and its association with HIV infection among MSM, a group with high HIV prevalence and incidence [18]. Only 30% of uncircumcised MSM showed some degree of willingness to be circumcised, a lower frequency than that reported in other countries [22].

Although previous studies reported that circumcision may offer some level of protection against HIV for MSM [23–25], the effect is expected to be modest given that, compared to heterosexual men, MSM engage in other sexual practices, such as RAI, which carry a high risk of infection. In fact, in this study, when we restricted the sample to men who do not engage in RAI, circumcision was significantly associated with decreased HIV prevalence. Our finding is consistent with results from some previous studies performed among MSM cohorts who predominantly engage in IAI [11, 13, 14]. However, other studies did not find a significant association even when data pertaining to IAI and RAI were considered [26].

Our results showed that more than 50% of the MSM engaged exclusively in insertive anal sex and/or oral sex, but not in RAI. This can be correlated with other findings in this group that 22% of the MSM were heterosexually identified [19]. This finding is consistent with other studies of Latin American MSM, where men who practice only insertive anal sex maintain a heterosexual identity [27].

In this study, being circumcised was not associated with a lower frequency of other STIs, even when the sample was restricted to those not engaging in RAI. Previous studies have found a reduction in transmission of *T. pallidum* among circumcised heterosexuals, although the same was not found among homosexuals [28], which is consistent with our results. Reduction in transmission of HBV and HCV among circumcised men is not clearly established. Previous studies reported circumcision as a risk factor for HBV and HCV infections in Bangladesh (among adults and children) and Egypt (among children) [29, 30], but this may be due to circumcisions being practiced by untrained health care providers, with the surgery being a route of transmission for the aforementioned infections. Regarding HPV, several studies established an overall reduction in genital HPV infection among circumcised men and also a reduction in the prevalence of high-risk HPV. Our results did not show a significant reduction in HPV prevalence among circumcised men who do not engage in RAI, even though there was a ten-point difference in the frequency. However, circumcision status in our study was significantly associated with a reduction of both the quantity of infection with HPV types and the quantity of infection with high-risk types, a finding that is consistent with previous reports [31].

Our data are subject to several limitations. Although our study shows an association between circumcision and HIV infection, the proportion of circumcised men in the sample was small (*n*=64), so a larger sample is needed to provide a reliable statistical comparison. Another limitation of our study is that circumcision was self-reported and not checked with clinical examination. Even when a previous validation study reported a high agreement between circumcision status and self-reports [32], sociocultural differences between MSM could make this finding not generalizable to our group. It should also be remembered that longitudinal randomized controlled trials are the optimal design to provide conclusive evidence for an association between circumcision and HIV transmission reduction. However, our data suggest that conducting such studies might not be feasible among MSM from Buenos Aires, because only a small proportion of MSM reported some grade of willingness to be circumcised.

These findings suggest that if circumcision was to be introduced as an HIV prevention strategy, education about the risks and benefits of circumcision would have to be developed and conducted prior to this intervention. Another finding from this study is that approximately 40% of MSM engage in RAI, so, even if circumcision could be accepted it would not be appropriate for HIV prevention of all MSM given that a large portion of them practice RAI [33, 34].

## Conclusions

Finally, a low frequency of circumcision was found among MSM in Argentina (13%) and, among those who were uncircumcised, only 30% expressed some grade of willingness to be circumcised. Even though no cases of HIV infection were detected among circumcised men who do not practice RAI, a larger sample of circumcised MSM is needed to provide a reliable statistical association between circumcision and protection from HIV infection.
